# An LLM-Based Framework for the Automatic Generation of SysML Models

**DOI:** 10.3390/s26165133

**Published:** 2026-08-13

**Authors:** Baoran An, Tao Lei, Guangtai Tian

**Affiliations:** 1 Institute of Computer Application, China Academy of Engineering Physics, Mianyang 621900, China; leitao25@gscaep.ac.cn; 2 School of Aeronautics and Astronautics, Sichuan University, Chengdu 610065, China; tiangt@scu.edu.cn

**Keywords:** SysML, large language model, model-based systems engineering, fine-tuning, prompt engineering, text-to-model

## Abstract

Model-based systems engineering (MBSE) takes Systems Modeling Language (SysML) as the industrial standard modeling language, yet cloud Large Language Model (LLM)-based SysML generation faces limited domain data, model hallucinations, high hardware cost and confidential data leakage risks. This paper builds a 914-sample SysML PlantUML corpus and proposes a fully offline lightweight framework based on Qwen2.5-Coder-7B-Instruct, integrating 4-bit NF4 Quantized Low-Rank Adaptation (QLoRA) fine-tuning, vector-free Jaccard same-diagram reference retrieval and a three-round syntax correction loop. PlantUML executes syntax parsing while Graphviz only renders layouts. Tested on 131 samples covering five structural and behavioral SysML v1 diagram types, the plain-prompt baseline achieves word-set semantic F1 of 52.94%, and the retrieval-enhanced variant lifts the zero-retry syntax pass rate from 92.37% to 99.28%, with F1 slightly dropping to 50.64%. Running fully local without cloud data transmission, this pipeline offers a privacy-safe lightweight solution for SysML PlantUML modeling and does not support SysML-exclusive requirement or parametric diagrams.

## 1. Introduction

The rising complexity of modern industrial systems drives the transformation from document-centric engineering to model-based systems engineering (MBSE). MBSE standardizes system design via formalized models, which improves design quality, shortens R&D cycles and realizes unified cross-department communication [1]. As the de facto industrial modeling standard, the systems modeling language (SysML) is an extended UML 2 dialect tailored for system architecture design, supporting multi-view modeling covering the full engineering lifecycle [2]. SysML standardizes the inheritance and reuse of enterprise proprietary system logic and has been widely adopted in automation and control industries.

Although OMG released SysML v2 in 2025, high industry migration costs have slowed its large-scale deployment. Most manufacturing and control enterprises still rely on mature SysML v1 workflow stacks [3]. This work focuses exclusively on automatic generation of SysML v1-compliant PlantUML code, supporting five mainstream structural and behavioral diagram types. SysML-native requirement diagrams and parametric diagrams are outside the scope of the dataset and model output. As a lightweight text-format intermediate tool, PlantUML converts natural language requirements into parseable structured code that can be rendered into graphical SysML models, transforming manual graphical modeling into code generation tasks suitable for large language model (LLM) automation [4].

Three prominent practical barriers hinder the wide adoption of MBSE in real engineering scenarios. First, rigorous SysML syntactic and semantic rules create a steep learning curve, requiring specialized training for model construction and interpretation [5]. Second, manual SysML modeling involves massive repetitive graphical formatting work, consuming engineers’ energy on format unification rather than core system logic analysis [6]. Third, mainstream commercial cloud LLM services force enterprises to upload confidential internal requirement descriptions to third-party servers, bringing irreversible risks of proprietary design information leakage. Against these practical pain points, this paper targets automated SysML v1 model generation from unstructured natural language requirements to reduce manual modeling overhead and lower the entry threshold of MBSE deployment. We construct a fully offline lightweight pipeline integrating customized prompt optimization, Jaccard word-set similarity offline same-diagram reference retrieval, 4-bit NF4 quantized low-rank adaptation (QLoRA) as a parameter-efficient fine-tuning (PEFT) scheme, and iterative PlantUML syntax error feedback correction, to jointly improve the syntactic validity and semantic matching quality of generated SysML PlantUML code.

Existing research on automated SysML generation can be split into two mainstream technical routes: rule-based transformation frameworks and transformer-based LLM generation methods. Representative rule-driven solutions are summarized in Table 1. Wan et al. [7] proposed the recursive object model (ROM) as an intermediate structured representation, and constructed hand-coded transformation rules to map ROM entities to block definition diagrams (BDDs), use-case diagrams and activity diagrams. Yang et al. [8] designed RNL2SysML for safety-critical cyber–physical systems, restricting input ambiguity via domain glossaries and deterministic rule conversion. Ouyang et al. [9] built template-based rule pipelines to map fixed sentence components to SysML graphical elements. Akundi et al. [10] combined lightweight machine learning with expert feedback to iteratively optimize transformation rule sets.

Horizontal comparative analysis reveals three inherent limitations shared by all rule-based methods: manual rule compilation heavily depends on senior domain experts; raw unstructured natural language must be manually standardized as preprocessing; hand-designed rule sets carry strong domain lock-in and weak generalization across heterogeneous system scenarios. Such drawbacks push academia to adopt pre-trained LLMs for automated SysML modeling.

Pre-trained transformers have achieved breakthrough performance on structured code generation tasks [11]. With the rapid expansion of model parameter scales, LLMs obtain powerful long-text comprehension and syntax-compliant structured output capabilities [12,13], shifting research focus away from traditional rule-based transformation. Current LLM-based SysML generation work falls into two categories: fine-tuning general-purpose foundation models and multi-agent collaborative modeling. Yang et al. [14] built a SysML generation framework based on a DeepSeek-7B encoder-only LLM. Bouamra et al. [15] constructed multi-agent template pipelines customized for SysML v2 specifications, which cannot be directly migrated to the SysML v1 workflow adopted in this paper.

Beyond narrow SysML-specific modeling literature, two branches of related research can be distinguished: generic cross-domain text-to-structured-artifact pipelines and targeted empirical LLM research for SysML behavioral model generation. For general system engineering scenarios, the work in [10] systematically summarizes universal automated text-to-model transformation workflows and core evaluation challenges for formal engineering artifacts, offering universal insights on requirement parsing and structured output constraint design applicable to MBSE tasks. For industrial IoT applications, ref. [16] builds natural-language-driven rule generation pipelines equipped with dedicated syntax validity verification modules, addressing identical structured output consistency and error filtering challenges encountered in SysML modeling.

The empirical study in [17] (the third literature specified by reviewers) focuses on LLM-based automatic generation of SysML behavioral diagrams and releases the only public ACM107 dataset containing 107 PlantUML-format behavior samples. It adopts rule-based checking to fix syntax defects and uses word-set F1 to measure semantic matching, revealing severe semantic hallucinations, especially for sequence diagrams. Nevertheless, this work only covers three categories of behavioral SysML diagrams without structural block definition diagrams, and it lacks core designs, including fully offline local deployment, category-matched reference retrieval augmentation, and a complete multi-round syntax correction loop. These cross-domain general pipelines and existing SysML empirical studies share core technical pain points, including unconstrained natural language input, mandatory domain-specific syntax rules and post-generation validity inspection, yet none integrate offline privacy-preserving deployment, diagram-matched retrieval context injection and iterative error feedback simultaneously, which further highlights the novelty and practical value of the fully offline lightweight retrieval-augmented solution proposed for confidential industrial SysML modeling tasks.

Universal LLM optimization paradigms, including zero/few-shot prompting, chain-of-thought reasoning and self-consistency calibration, effectively boost logical reasoning and structured generation stability [18]. In requirement engineering scenarios, role assignment and context reference examples bring obvious performance gains, while context-aware prompt templates improve output consistency and task efficiency [19,20].

Despite the advantages of LLM pipelines over rule-based schemes, existing LLM-driven SysML modeling research still faces three unaddressed critical gaps summarized in recent domain surveys, ordered by industrial practical priority:

1. Data security risk of cloud LLMs: All mainstream high-performance foundation models only provide cloud Application Programming Interface (API) access, forcing enterprises to upload confidential requirement texts and introducing irreversible proprietary design leakage risks. Few open-source schemes support fully offline local deployment.
2. Shortage of high-quality domain corpus: Public labeled SysML PlantUML datasets are extremely scarce, fundamentally limiting the upper bound of model generation performance [21,22]. The only public ACM107 dataset suffers from a small sample volume, incomplete diagram coverage and insufficient quality filtering.
3. Lack of SysML-specific hallucination suppression strategies: Native LLM semantic and syntactic hallucinations cannot be fully eliminated, while general error mitigation methods are rarely adapted to strict SysML syntax constraints [23,24].

To fill the above multi-dimensional research and practical gaps, this paper proposes a fully offline lightweight generation pipeline combining four core modules: customized constraint prompt optimization, offline Jaccard word-set similarity same-diagram reference retrieval, 4-bit NF4 QLoRA PEFT fine-tuning, and a multi-round PlantUML syntax error feedback correction loop [25,26]. We adopt the open-source Qwen2.5-Coder-7B-Instruct model to support complete local Graphics Processing Unit (GPU) deployment and eliminate reliance on cloud model services.

All experiments are conducted on a self-constructed strictly filtered industrial corpus containing 914 valid pairs of natural language requirements and standard renderable SysML PlantUML code. The corpus is split into a 783-sample training set and a 131-sample held-out test set. All raw samples are filtered via PlantUML.jar syntax inspection to discard unparseable code snippets. Graphviz is only used for final graphical rendering and does not participate in dataset quality screening. Comparative ablation experiments carried out on the unified test set verify that the offline retrieval module significantly improves native one-pass syntax compliance at the cost of a slight decline in semantic matching performance. The whole pipeline runs fully locally on a single NVIDIA A10 GPU without uploading confidential requirement data to third-party platforms, fundamentally eliminating data leakage hazards for industrial MBSE modeling tasks.

The core contributions of this work are summarized as follows:

1. A high-quality manually filtered SysML v1 PlantUML paired corpus with 914 valid samples covering five mainstream diagram types and 16 industrial domains is constructed, which outperforms the only public ACM107 dataset in scale, category coverage and syntax validity.
2. A fully offline lightweight generation framework based on 4-bit NF4 QLoRA fine-tuning is proposed, requiring only a single consumer-grade GPU for local deployment and avoiding confidential data transmission to cloud servers.
3. A two-stage prompt ablation optimization scheme is designed to obtain the lightweight standardized baseline template Type1_SimpleRule.
4. An offline Jaccard same-diagram retrieval module is integrated to dynamically inject matching reference examples for boosting native one-pass syntax compliance without vector databases or embedding models.
5. A closed-loop PlantUML syntax correction workflow with up to three regeneration retries is embedded in inference, which significantly improves the final proportion of renderable valid SysML models.

## 2. Methodology: Optimization of LLM for SysML Model Generation

**Figure 1:**
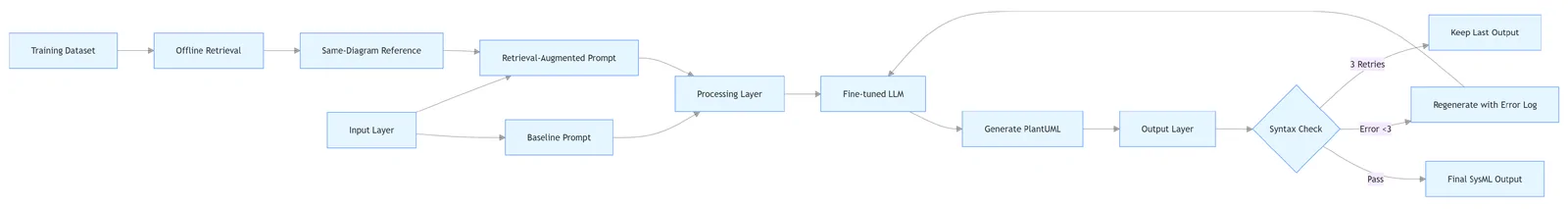
Overall workflow of the proposed fully offline generation framework.

This section presents a fully offline and lightweight framework for transforming unstructured natural-language system requirements into standardized SysML v1 PlantUML models. The complete pipeline is illustrated in Figure 1, consisting of a three-layer sequential generation architecture and an independent offline retrieval branch specifically designed for inference-stage enhancement.

The overall workflow includes an input layer, a model processing layer, and an output correction layer. At the input layer, domain-customized structured prompt templates are constructed to standardize requirement inputs. For retrieval-augmented inference, category-consistent PlantUML reference snippets are dynamically retrieved from the training dataset and embedded into the prompt context. The processing layer adopts a domain-adapted lightweight LLM as the core module for PlantUML code generation. At the output layer, automated syntax validation and multi-round error feedback regeneration are performed to guarantee parseable and renderable model outputs.

To quantitatively investigate the effects of reference augmentation, two prompt settings are designed for ablation comparison: a baseline prompt without external reference support, and an enhanced prompt integrated with category-matched SysML modeling examples.

A dedicated two-stage validation workflow is adopted for post-generation quality inspection, where PlantUML.jar (v1.2026.6) serves as the sole syntax validator and Graphviz (v15.1.0) only undertakes graphical layout rendering. Specifically, PlantUML.jar strictly detects syntactic defects including unmatched brackets, undefined stereotypes, and invalid SysML keywords, while Graphviz does not participate in error judgment or validity screening. Once syntax faults are identified, the parsed error logs are fed back to the original prompt to support iterative regeneration, with a maximum of three correction rounds to avoid infinite iteration.

Crucially, the offline retrieval module is enabled only during inference. The entire fine-tuning process relies purely on baseline prompts without any retrieved examples, ensuring no information leakage between training and inference. Moreover, the retrieval mechanism strictly restricts candidate samples to those with identical SysML diagram types, ensuring category-consistent structural guidance.

Benefiting from the task-specific fine-tuning and retrieval-augmented correction pipeline, the whole system supports fully local deployment on a single consumer-grade GPU. No requirement data is uploaded to third-party cloud services, fundamentally eliminating confidential information leakage risks in industrial MBSE modeling scenarios.

### 2.1. Dataset Analysis and Data Cleaning

Data quality constitutes a fundamental prerequisite for stable domain-specific LLM training and reliable structured code generation [21,22]. This section details the construction pipeline, cleaning criteria, statistical characteristics, and inherent limitations of the custom SysML v1 PlantUML corpus built for this study.

The raw corpus is derived from a publicly available PlantUML modeling dataset on HuggingFace, originally stored in CSV format and processed using the Pandas library (v3.0.2). Unlike conventional text–code paired datasets, each sample is annotated with multi-dimensional labels, including industrial domain, SysML diagram type, modeling complexity grade, natural language requirement, ground-truth PlantUML code, and binary parsing validity status. All samples correspond to independent industrial system design cases with complete labeling and renderable model structures.

Systematic data denoising and filtering are conducted to adapt the raw corpus to standardized SysML v1 modeling tasks. Redundant prefix tags and irrelevant descriptive sentences in requirement texts are removed to preserve concise and complete design specifications. All samples undergo strict syntax validation exclusively via PlantUML.jar (v1.2026.6), which detects structural defects including unmatched brackets, undefined stereotypes, and invalid SysML keywords. Graphviz (v15.1.0) is only used for final graphical rendering and does not participate in any data cleaning or validity judgment. Samples with unparseable, incomplete, or non-compliant PlantUML structures are directly eliminated. After this two-stage screening, a total of 914 high-quality requirement–code paired samples are retained, all of which pass automatic syntax verification and graphical rendering tests.

The cleaned corpus is divided into a training set of 783 samples and a held-out test set of 131 samples following a fixed 85%/15% partition ratio. A consistent random seed (2026520) is adopted to ensure reproducible data splitting for all ablation and comparative experiments.

A medium-complexity use-case diagram sample from the medical equipment domain is selected to demonstrate the standardized data format. The corresponding natural language requirement is presented as follows:

After completing a patient’s X-ray using the digital imaging equipment, the
radiologic technologist initiates the medical record archiving process.
The machine automatically compiles scan images and equipment performance
logs into a single digital file. The technologist selects the Archive
option on the equipment’s control panel. The system validates the patient
identification number and securely transmits compiled records to the
central hospital storage server. Upon successful transmission, the
equipment display outputs a confirmation message. The digital medical
record is permanently saved and accessible for subsequent physician
review.

**Figure 2:**
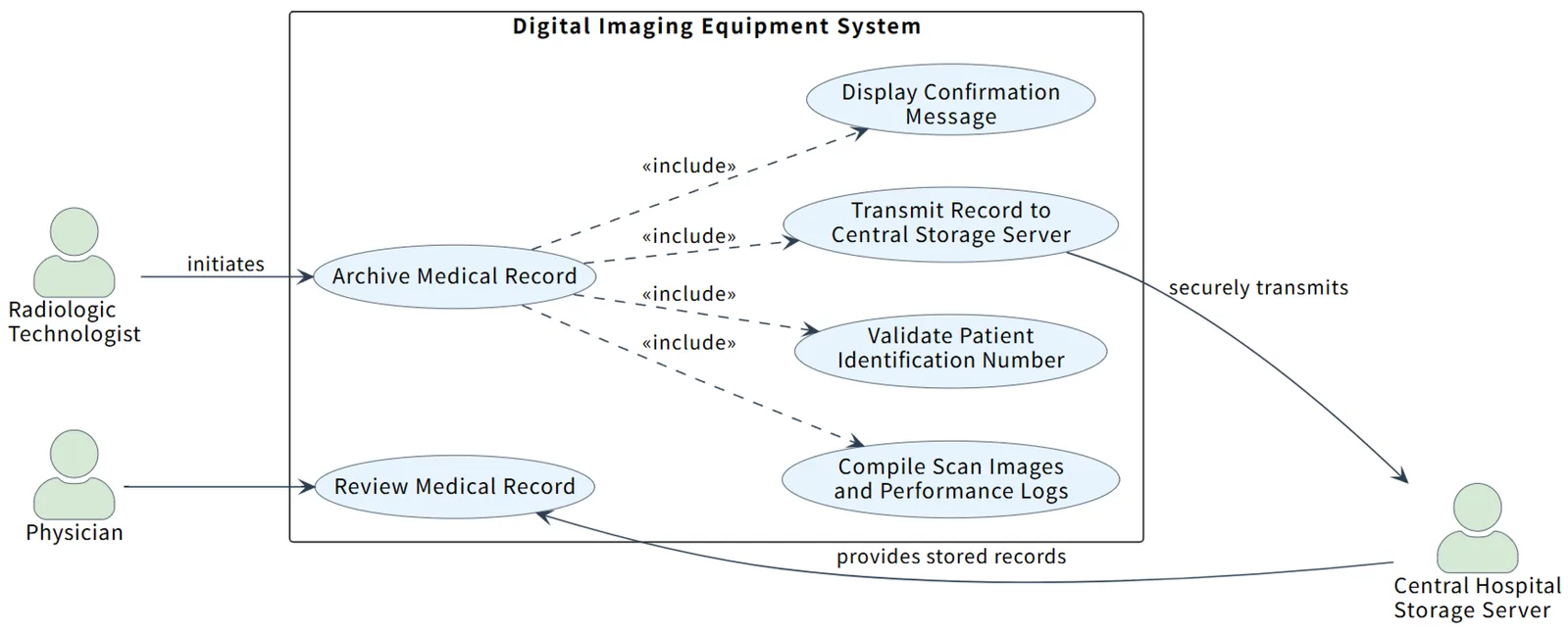
Renderable PlantUML code corresponding to the medical use-case sample.

The standard SysML v1 PlantUML code for this case is illustrated in Figure 2, which strictly follows industrial modeling specifications regarding system participants, interactive relations, and data transmission logic.

The constructed corpus covers 16 distinct industrial domains, including new energy power systems, intelligent vehicle control, medical devices, automated production lines, financial service platforms, IoT systems, and logistics management. Such cross-domain coverage enhances the model’s generalization capability for diverse system engineering modeling scenarios.

**Figure 3:**
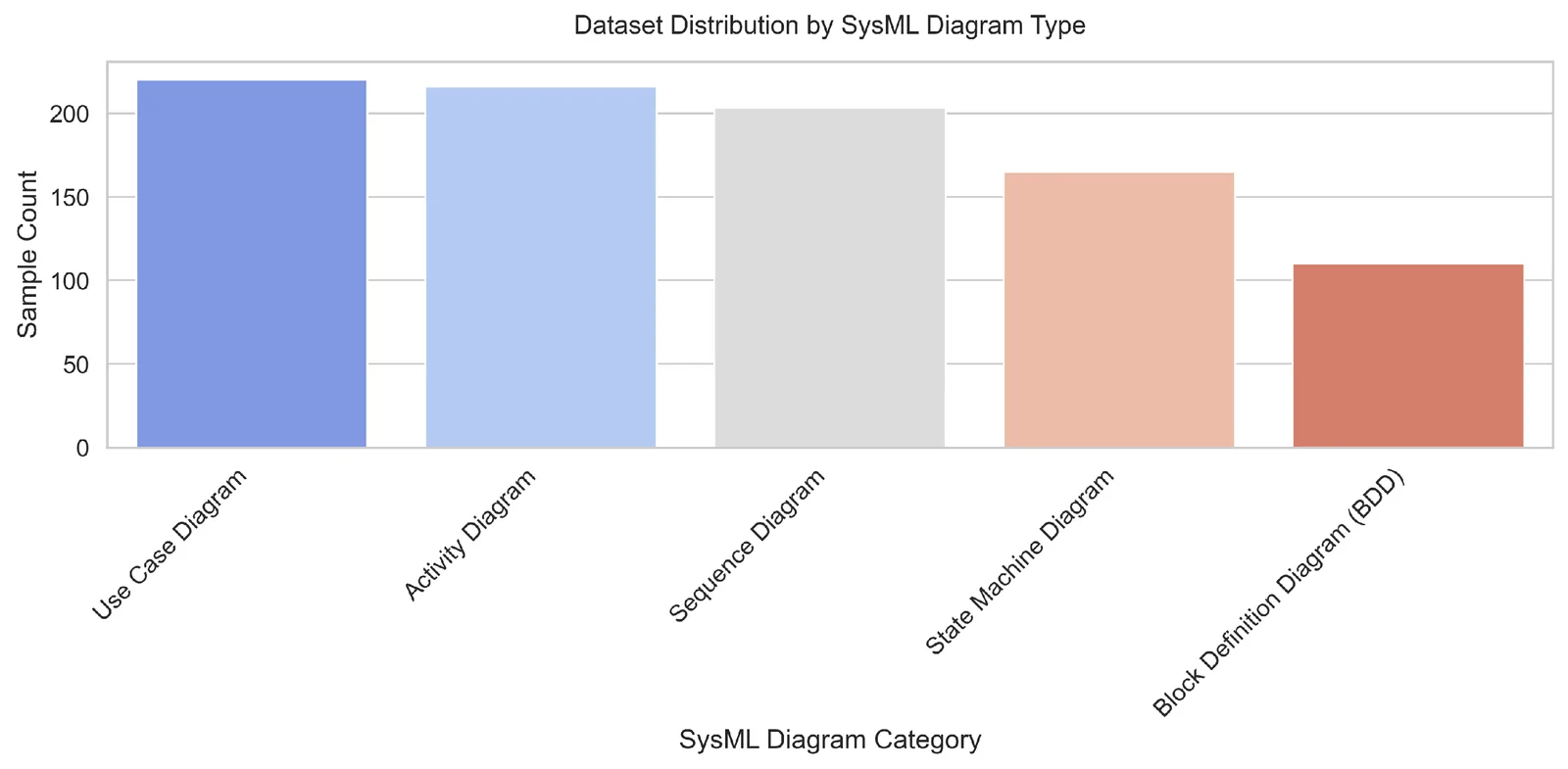
Sample distribution of five standard SysML v1 diagram types.

In terms of diagram diversity, the dataset supports five mainstream SysML v1 diagram types: block definition diagrams (BDDs), use-case diagrams, activity diagrams, sequence diagrams, and state machine diagrams. All ground-truth PlantUML codes strictly comply with SysML v1 syntax and semantics rather than generic UML rules. SysML-specific requirement diagrams and parametric diagrams are excluded from the current dataset scope. The sample distribution across five diagram categories is shown in Figure 3.

**Figure 4:**
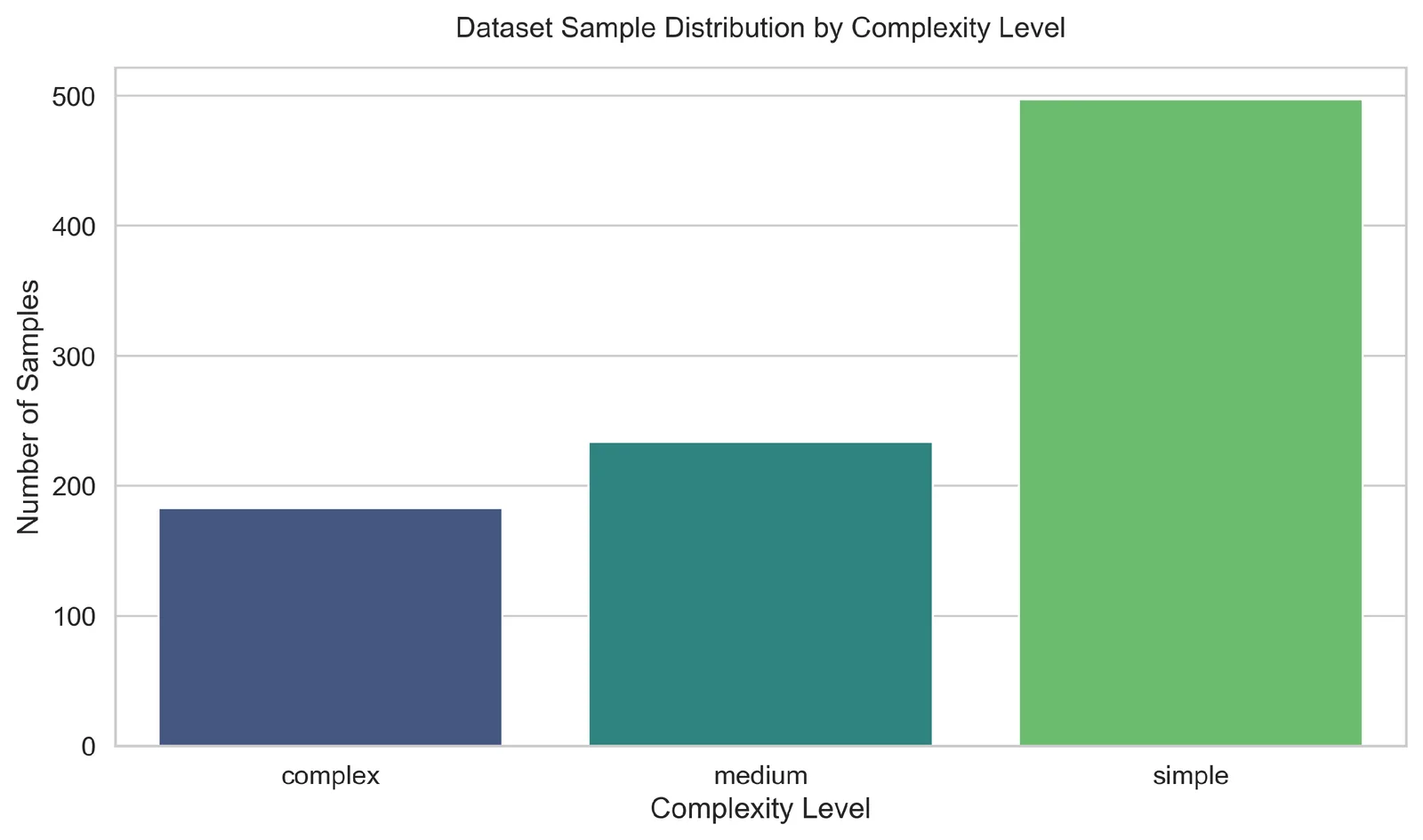
Sample distribution grouped by modeling complexity level.

All samples are manually categorized into three complexity levels: simple (497 samples), medium (234 samples), and complex (183 samples). As visualized in Figure 4, samples with multi-branch nonlinear system logic account for a relatively small proportion, limiting model performance on highly complex system modeling tasks.

**Figure 5:**
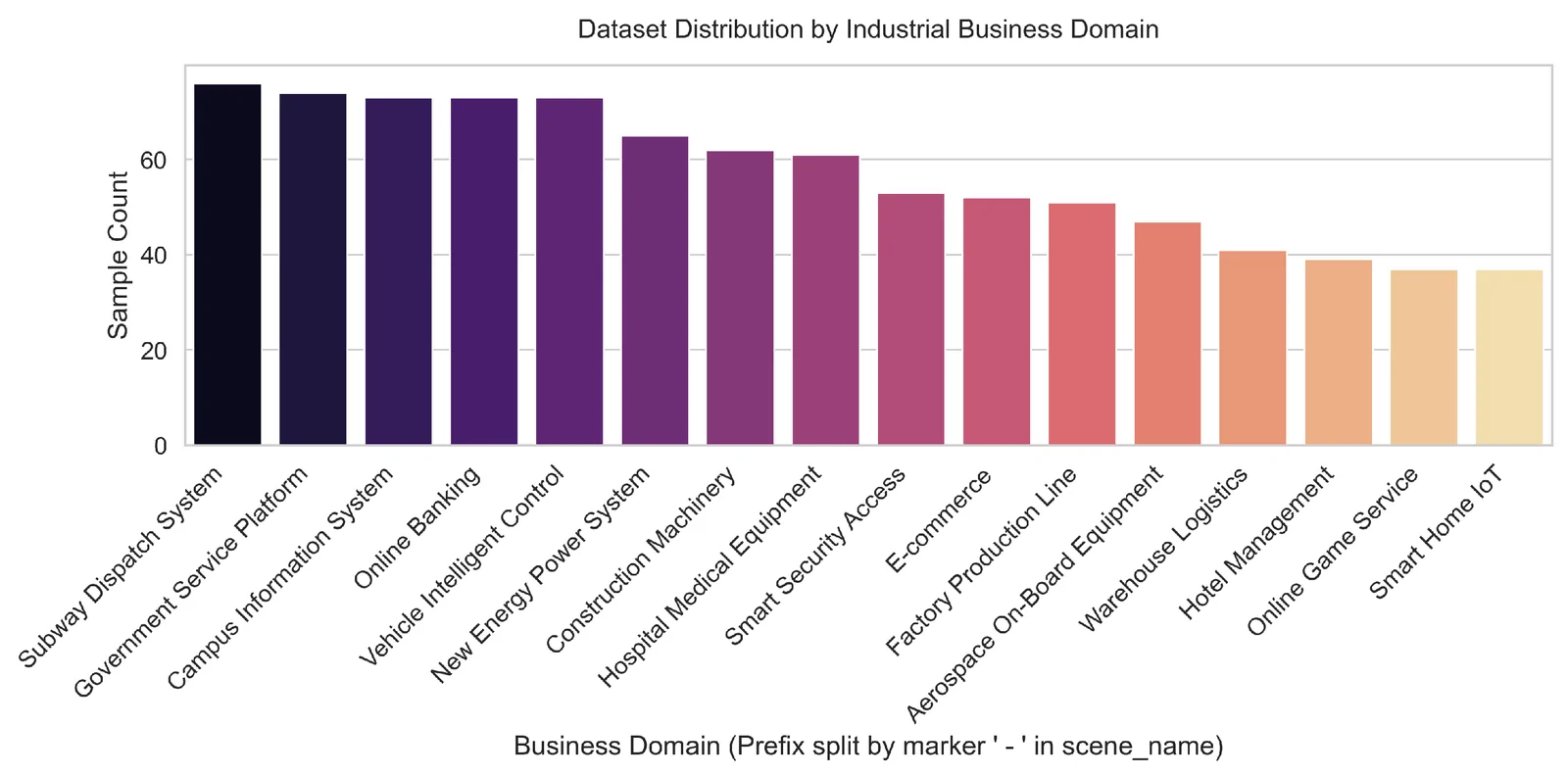
Sample distribution across industrial business domains.

The sample distribution across all industrial domains is demonstrated in Figure 5.

**Figure 6:**
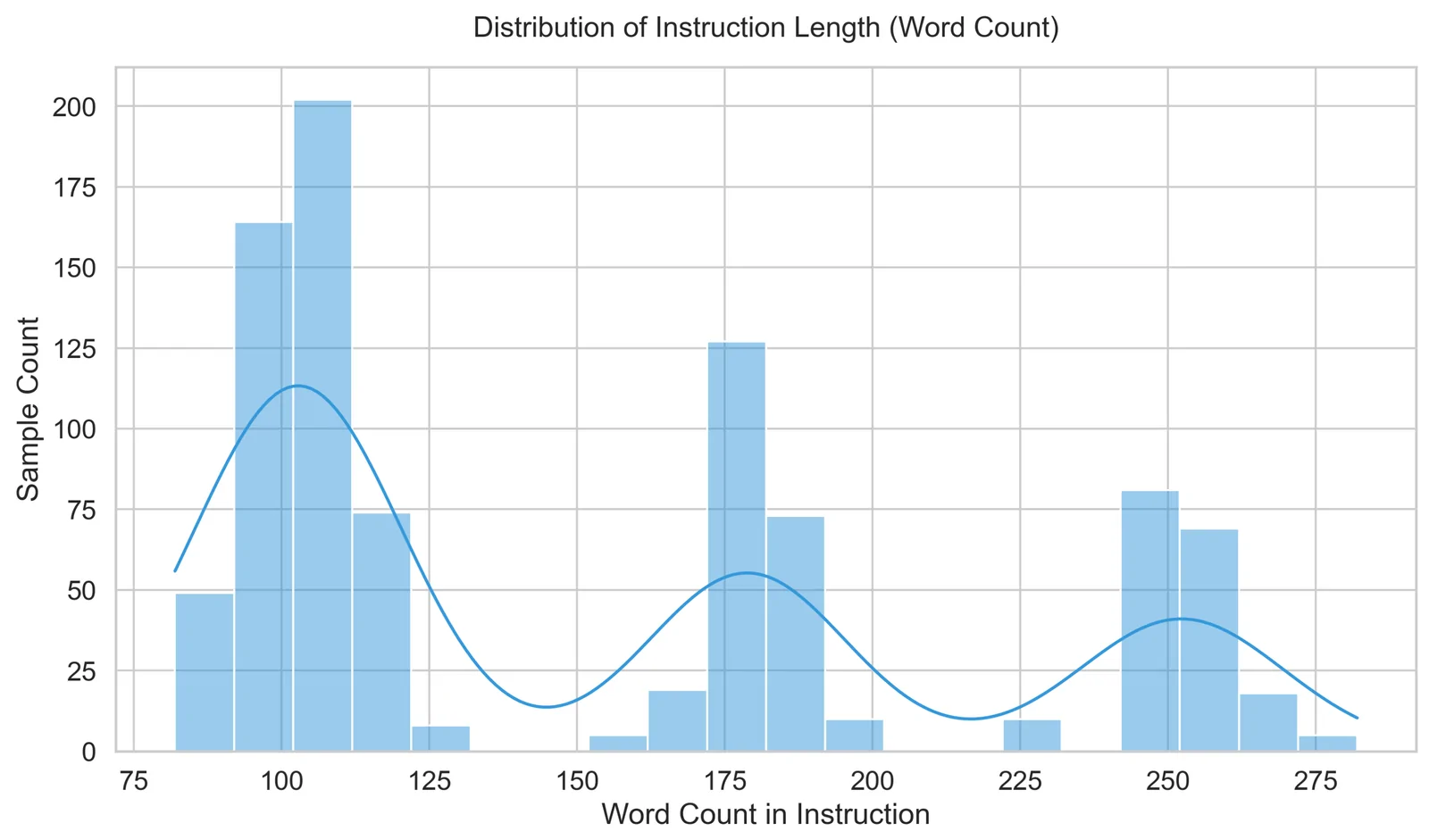
Length distribution of natural language requirement instructions.

Quantitative statistical analysis is performed on both input requirement texts and output PlantUML code. The natural language requirements achieve an average word count of 152.26 and an average character count of 1054.55, with the length distribution shown in Figure 6.

**Figure 7:**
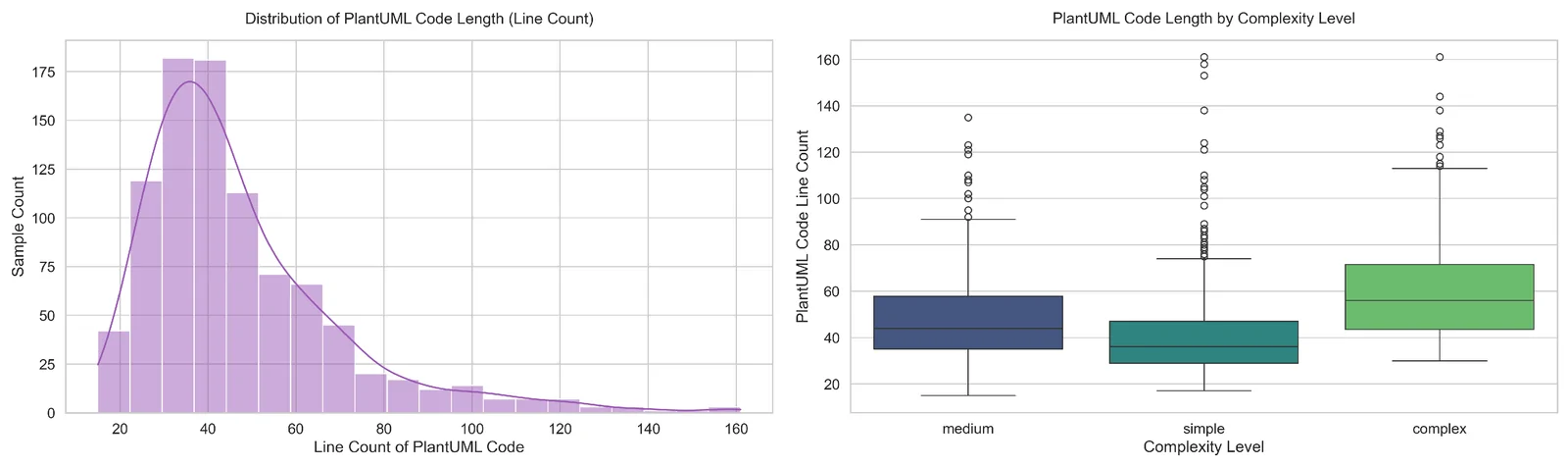
(**Left**): length distribution of ground-truth SysML PlantUML code. (**Right**): code line count distribution by modeling complexity.

Each ground-truth PlantUML snippet averages 47.36 lines and 1649.57 characters. Figure 7 presents the overall code length distribution and complexity-based code line statistics. Complex modeling cases contain more system modules and interactive relations, resulting in longer structured code.

**Figure 8:**
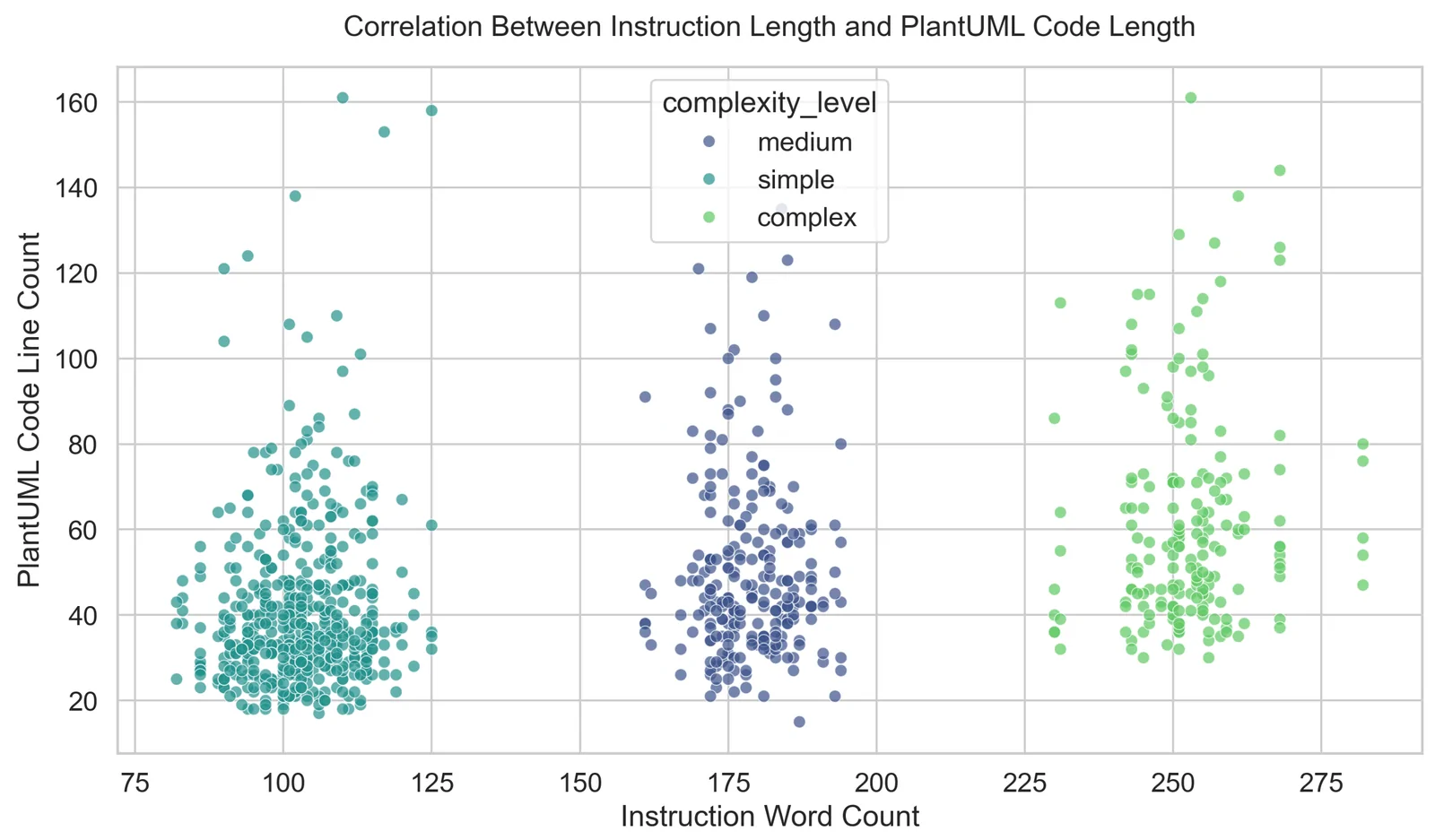
Correlation between requirement text length and PlantUML code scale.

Pearson correlation analysis is further conducted to explore the association between requirement text length and model scale. The overall correlation coefficient is 0.3655 with statistical significance, indicating a weak positive linear relationship. After grouping by complexity level, the correlation further decreases, demonstrating that lengthy textual descriptions do not inherently represent complex system logic. The corresponding scatter plot is provided in Figure 8.

Table 2 summarizes the core statistical attributes of the finalized corpus.

Compared with the publicly available SysML corpus (ACM107), the present dataset achieves larger sample volume, broader diagram coverage, and stricter syntax filtering. Nevertheless, the limited number of nonlinear multi-branch modeling samples remains a recognized limitation, which is further analyzed in the Discussion section. To support reproducible research, all dataset construction rules, sample partitioning protocols, and filtering standards are fully disclosed in this manuscript.

### 2.2. Prompt Engineering and Two-Stage Ablation Optimization

Prompt engineering acts as an economical lightweight optimization used to boost domain-specific generation performance of large language models [27]. Widely adopted prompt paradigms each carry distinct merits and limitations. Few-shot prompting constrains output formats and helps models to capture domain patterns, yet fixed manual demonstration samples may introduce generation bias and consume extra context window capacity. Role prompting steers LLMs to produce domain-targeted content with low overhead, but it easily results in rigid, formulaic outputs. This work integrates professional role definition and standardized format constraints to build customized prompts dedicated to SysML modeling tasks.

Two augmentation mechanisms should be distinguished at the outset. The proposed offline framework abandons conventional embedding- and vector-database-based retrieval-augmented generation. Instead, an independent offline matching branch built on Jaccard word-set similarity dynamically appends matched modeling snippets to base prompts. The retrieval-enhanced prompt variant is elaborated in a subsequent dedicated retrieval section. This section only investigates static baseline prompts without supplementary reference examples.

Unlike generic code generation tasks where minor syntax defects have limited functional impact, PlantUML modeling code must follow strict unified syntax rules to render complete graphical SysML diagrams. To reduce unparseable invalid outputs, we design baseline prompt templates via a two-stage ablation filtering pipeline rather than simply stacking scattered constraint clauses. The optimized template serves as a lightweight foundational component of the overall offline SysML generation framework, guiding the fine-tuned LLM to learn standardized output rules and raise the proportion of syntactically valid PlantUML code in native single-pass generation.

Two quantitative metrics are adopted to evaluate prompt performance: word-set semantic F1 and PlantUML parse pass rate. The word-set semantic F1 quantifies semantic alignment between generated PlantUML and ground-truth SysML models. For each prediction and reference pair, we first extract code enclosed between the tags “@startuml” and “@enduml”, strip all punctuation, convert all characters to lowercase, and partition text into unique word sets without weighting term frequency. The corresponding calculation formulas are defined as

$$\mathrm{Precision}=\frac{\left|A\cap B\right|}{\left|A\right|},\;Recall=\frac{\left|A\cap B\right|}{\left|B\right|},\;F1=\frac{2\times \mathrm{Precision}\times Recall}{\mathrm{Precision}+Recall}$$

where *A* denotes the unique word set of generated code and *B* represents the word set of ground-truth code. For the PlantUML parse pass rate, validity judgment is performed solely by PlantUML.jar. Graphviz only undertakes graphical layout rendering and does not participate in syntax inspection.

All prompt ablation experiments adopt a fixed small test subset of 20 samples extracted from the full 131-sample held-out test set, balancing evaluation efficiency and consistent comparison conditions across all prompt variants.

Seven prompt variants with different constraint combinations are constructed for primary screening in the first ablation stage: plain baseline templates, dialogue-oriented templates, minimal simplified templates, rigid format constraint templates, chain-of-thought reasoning templates, expert role templates, and full composite constraint templates. Full experimental results for all seven schemes are summarized in Table 3, where the parse pass rate records the share of renderable valid PlantUML generated in a single forward pass over the 20-sample subset.

As shown in Table 3, Type4_FormatRule achieves the highest native parse pass rate of 75.00% under strict format restrictions, while Type6_ExpertRole delivers the optimal word-set semantic F1 of 0.4348 by assigning a professional system modeling engineer identity. These two templates with outstanding single-dimension performance are selected as the foundation for secondary optimization.

In the second refinement stage, two revised prompt templates are reconstructed based on the two screened baseline schemes above. Both variants embed a unified role definition that directs the LLM to act as a senior SysML v1 modeling engineer specializing in standardized PlantUML design. Comparative ablation tests are conducted on the identical 20-sample test subset, with complete results reported in Table 4.

Type1_SimpleRule inherits rigid format constraints from Type4_FormatRule and adds unified mandatory output rules to eliminate redundant explanatory text, blank lines, and non-standard symbols. Type2_ExpertDiagramRule retains the expert reasoning logic of Type6_ExpertRole and embeds diagram-specific constraint rules for all five SysML categories to avoid extraneous irrelevant system elements. Universal grammatical and modeling constraints shared by both refined templates are listed below:

- All generated code must start with “@startuml” on the first line and terminate with “@enduml” on the final line.
- Only pure PlantUML source code can be output. Supplementary explanations, standalone comments, quotation marks, Markdown tags, and redundant blank lines are prohibited.
- All outputs must conform to official standard PlantUML syntax without self-defined grammar, ensuring full parsing compatibility with PlantUML.jar.
- Three standardized SysML modeling requirements are enforced: consistency (no logical contradictions between model code and input requirements), traceability (each graphical element maps one-to-one to original requirement descriptions), and verifiability (hierarchical code structure supporting automatic tool inspection and manual audit).

Table 4 indicates that Type1_SimpleRule attains a word-set semantic F1 of 0.4378 while maintaining a 75.00% native parse pass rate, outperforming Type2_ExpertDiagramRule in both semantic matching and syntax compliance. In contrast, Type2_ExpertDiagramRule only reaches an F1 of 0.3881, with its parse pass rate declining to 60.00%. Excessive diagram-specific hard constraints cause the model to generate overly conservative outputs, omitting core system entities and association relations extracted from input requirements and degrading overall modeling quality.

The two-stage ablation results demonstrate that the lightweight unified format template Type1_SimpleRule achieves the optimal balance between semantic matching capacity and native syntax compliance. This template is fixed as the baseline prompt adopted uniformly across all subsequent QLoRA fine-tuning and inference experiments. The full text of the optimal Type1_SimpleRule template is provided below:

Generate standard SysML PlantUML source code for diagram name:
 {full_diagram_name} per user instruction: {instruction}
# Strict Generation Rules
1. Code must strictly start with @startuml and end with @enduml as
the first and last line
2. Output ONLY raw PlantUML code, zero extra text, explanations,
notes, comments, markdown symbols or any quotation marks
3. Follow official SysML PlantUML syntax, fully compatible with
PlantUML parsing engine
4. No leading/trailing blank lines outside the uml block
PlantUML Code:

### 2.3. LoRA Fine-Tune

Cloud general large language models achieve competitive performance on universal code generation tasks [17], yet uploading confidential industrial system requirements to third-party servers brings severe data leakage risks and fails to meet industrial data security standards. To support fully offline local SysML modeling without reliance on cloud services, this paper adopts lightweight parameter-efficient fine-tuning (PEFT) based on open-source small-scale large language models. The domain model fine-tuned locally lowers hardware deployment barriers and achieves better performance than general cloud large language models on professional SysML PlantUML generation tasks, even though general foundation models such as DeepSeek obtain promising results on generic code benchmarks [28].

Parameter-efficient fine-tuning (PEFT) has become the mainstream lightweight training paradigm for large language models. As a classic PEFT implementation, Low-Rank Adaptation (LoRA) balances training computational overhead and downstream modeling performance. It freezes all pre-trained backbone parameters and only optimizes tiny low-rank decomposition matrices, where the number of trainable parameters accounts for less than one percent of the total model parameters. LoRA can reach or even exceed the performance of full fine-tuning on domain adaptation tasks, which perfectly matches the fully offline SysML modeling scenario proposed in this paper [29]. Its core mechanism inserts two trainable low-rank matrices into transformer attention projection layers to learn domain-specific SysML modeling knowledge [30,31].

For the target attention projection layers q_proj and v_proj of Qwen2.5-Coder-7B-Instruct, the input and output hidden dimension satisfies $d_{\mathrm{in}}=d_{\mathrm{out}}=7168$, and the LoRA rank hyperparameter is set to r=8. The two decomposition matrices follow the standard LoRA formal definition:

$$\begin{array}{cc} A & \in R^{r\times d_{\mathrm{in}}}=R^{8\times 7168},\\ B & \in R^{d_{\mathrm{out}}\times r}=R^{7168\times 8}. \end{array}$$

Matrix A maps the original high-dimensional input features down to low-dimensional latent space, and matrix B projects the compressed latent low-dimensional features back to the original high-dimensional output space, forming the incremental weight term ΔW=BA. The forward propagation formula of linear layers integrated with LoRA is defined as

$$h=Wx+\frac{\alpha }{r}\mathrm{BA}x$$

where the scaling factor α=16. This paper adopts 4-bit normalized floating-point quantization (NF4) quantized LoRA (QLoRA), which combines weight quantization and LoRA fine-tuning to drastically reduce training memory occupation with negligible degradation of generation performance [32]. The PagedAdamW 8-bit optimizer is adopted to stabilize model convergence during training [33]. The complete QLoRA supervised fine-tuning pipeline is divided into four independent stages, named Official QLoRA-Supervised Fine-Tuning (SFT) Config.

#### 2.3.1. Dataset Preparation and Partitioning

All training procedures run completely offline without Internet access. Samples containing unparseable PlantUML code filtered out by PlantUML.jar are discarded. Graphviz only performs graphical layout rendering and does not participate in data cleaning screening. The 914 valid requirement and PlantUML paired samples are split into a training set of 783 entries and a held-out test set of 131 entries at a fixed ratio of 8.5 to 1.5. A fixed random seed 2026520 is adopted to guarantee repeatable experimental division [34]. A subset of 20 samples extracted from the test set is applied to the prompt ablation experiments described in the previous section. The overall corpus scale is suitable for small-data domain adaptation targeting SysML modeling tasks [35].

#### 2.3.2. SFT Dialogue Sample Construction

According to the optimal baseline template Type1_SimpleRule screened in Section 2.2, standardized dialogue training samples are constructed. The system prompt defines the model identity as a professional engineer specializing in SysML v1 standardized PlantUML design. The maximum input sequence length is limited to 1024 tokens to control memory overhead. Each training sample is organized in the format of system role description, natural language design requirement and ground-truth SysML PlantUML code. All samples are processed via the official Qwen chat template and converted into Hugging Face Dataset format for subsequent supervised training.

#### 2.3.3. Hyperparameter Configuration

The base model selected in this work is Qwen2.5-Coder-7B-Instruct, which possesses outstanding capability for structured code generation tasks [28,36]. All training processes are executed on a single NVIDIA A10 GPU (NVIDIA Corporation, Santa Clara, CA, USA) equipped with 23 GB Video Random Access Memory (VRAM). Gradient checkpointing and 4-bit NF4 weight quantization are enabled simultaneously to cut down memory usage, so the whole training pipeline can run on a single consumer-grade GPU without high-end computing clusters.

Core hyperparameters of the LoRA module:

1. LoRA rank *r*: 8;
2. lora-alpha scaling factor: 16;
3. lora-dropout: 0.05;
4. Trainable target attention layers: q_proj, v_proj.

Global training hyperparameters for SFT:

1. Training epochs: 4;
2. Per-device batch size: 2, gradient accumulation steps: 4 (effective batch size 8);
3. Initial learning rate: $2\times 10^{-4}$;
4. Warmup ratio: 0.05, learning rate scheduler: cosine decay;
5. Weight decay: 0.01, maximum gradient norm: 1.0;
6. Optimizer: PagedAdamW 8-bit.

All transformer backbone layers are fully frozen throughout training. Only the low-rank decomposition matrices inside q_proj and v_proj are updated and optimized.

#### 2.3.4. Fine-Tuning Execution and Convergence Analysis

**Figure 9:**
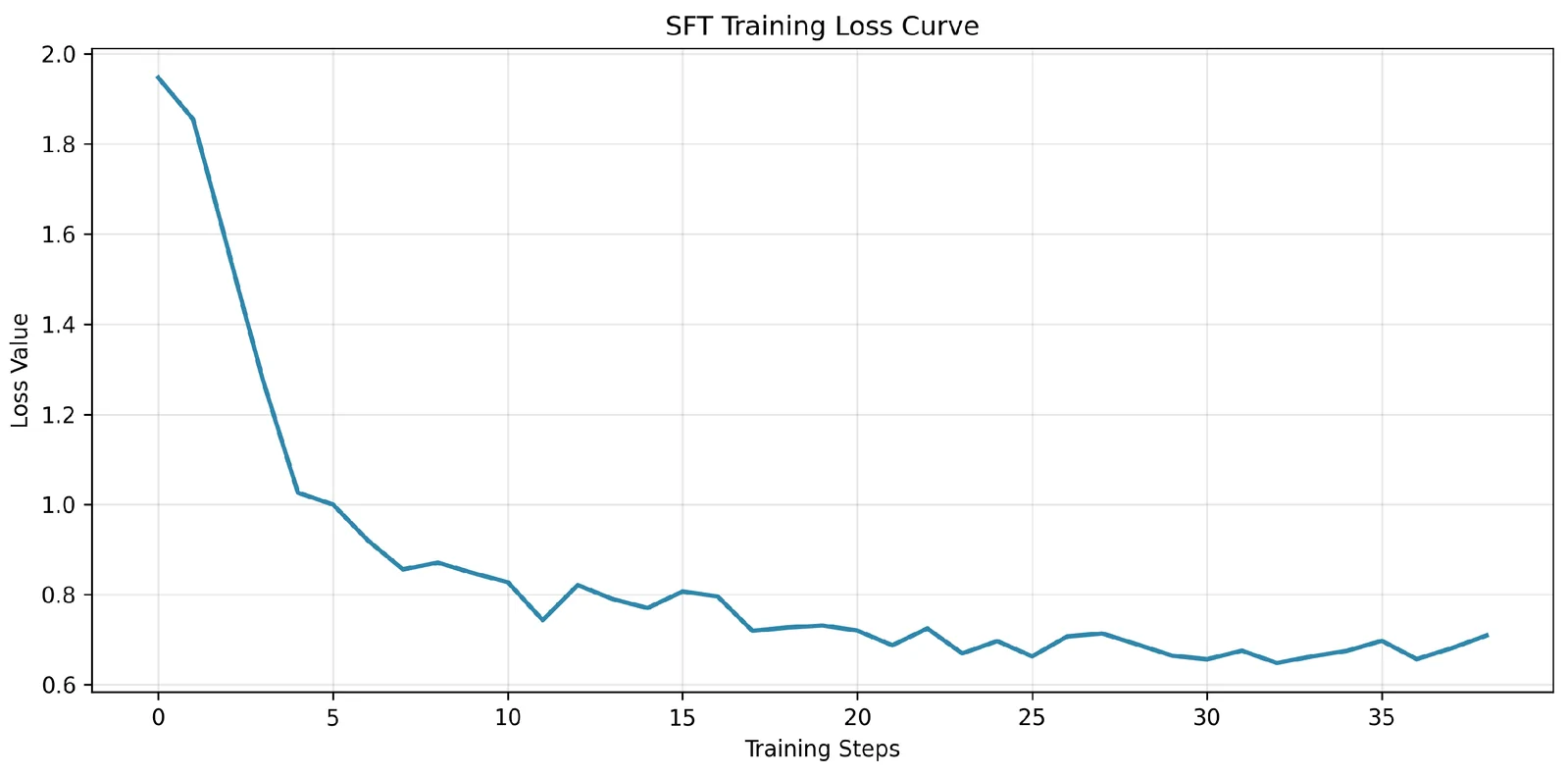
Training loss curve of QLoRA SFT.

Training loss values are recorded every 10 update steps across four full training epochs. The loss fluctuates within the range from 0.64 to 0.71 along cumulative training steps, rather than decreasing monotonically. Such oscillation originates from insufficient training samples with multi-branch nonlinear system logic, which limits the model’s generalization performance on high-complexity SysML modeling tasks. After training converges, the trained LoRA adapter weights are saved locally for follow-up inference ablation comparison experiments. The overall downward trend of the loss curve proves that the model effectively learns standard SysML v1 PlantUML syntax rules and the mapping relation between natural language design requirements and graphical model elements, as illustrated in Figure 9.

### 2.4. Offline In-Context Example Retrieval Module

To improve the syntactic standardization and structural rationality of generated SysML v1 PlantUML code, this paper designs an offline example retrieval branch attached to the input layer of the overall generation pipeline.

The retrieval knowledge base is exclusively built from the 783 training samples, with all 131 held-out test samples excluded to completely eliminate cross-set data leakage during evaluation. Each storage entry stores paired natural language requirements and validated SysML PlantUML code for subsequent similarity matching and contextual reference.

This work adopts lightweight Jaccard word-set similarity to implement offline lexical matching. Unlike embedding-based vector retrieval pipelines, this overlap-driven metric requires no additional pre-trained encoders or dedicated vector databases, and it avoids indexing bias introduced by small-scale domain-specific datasets. The Jaccard similarity calculation formula is defined as

$$J\left(A,B\right)=\frac{\left|A\cap B\right|}{\left|A\cup B\right|}$$

where *A* and *B* denote unique word sets extracted from test requirements and training-set requirements, respectively. Consistent with the preprocessing pipeline for word-set F1 evaluation, all texts undergo unified normalization: punctuation removal, lowercase conversion, and extraction of distinct vocabulary without frequency weighting.

A SysML-task-specific hard constraint is enforced during retrieval: only training samples sharing an identical SysML diagram type with the target test case are treated as matching candidates. Different SysML diagram categories follow independent rules for element definition and relational association, making cross-type reference examples semantically inconsistent. Among all same-category candidates, the training sample with maximum Jaccard similarity is selected as the supporting reference snippet.

The retrieved PlantUML reference content is concatenated after the optimized Type1_SimpleRule baseline template to form retrieval-augmented inference prompts, following the fixed format shown below:

Reference valid {diagram type} PlantUML Example (similar requirement):
{best reference PlantUML code}
You must strictly learn and follow the syntax, element structure and
keyword usage of the above reference example.

It is critical to clarify that dynamically matched retrieval-augmented prompts are only activated at inference time. The full QLoRA fine-tuning process solely adopts the plain Type1_SimpleRule template without any attached reference examples. This diagram-type-constrained context enhancement strategy creates two independent comparative branches for follow-up ablation experiments, enabling quantitative measurement of performance gains brought by injecting matching reference examples into SysML modeling generation.

Overall, this lightweight offline retrieval module avoids the overhead and leakage risks of embedding vector databases, while category-matching constraints ensure semantically consistent contextual guidance to boost syntactic compliance and logical rationality of generated SysML PlantUML outputs.

### 2.5. Model Inference and Syntax Verification

The 4-bit NF4 quantized QLoRA fine-tuned Qwen2.5-Coder-7B-Instruct model is deployed for fully offline local inference. A dedicated error-feedback closed-loop correction pipeline is constructed for SysML v1 PlantUML generation. Identical inference hyperparameters are adopted across all ablation groups to guarantee fair comparative evaluation. The core generation hyperparameters are listed as follows:

- Maximum new tokens: 1200, temperature: 0.08;
- Top-p: 0.92, top-k: 20, repetition penalty: 1.06;
- Upper limit of syntax correction retries: 3.

Two independent inference variants are designed for controlled ablation comparison: a baseline variant using plain prompts without supplementary reference examples and a retrieval-augmented variant that appends category-consistent SysML PlantUML reference snippets matched via Jaccard similarity. The complete generation and correction workflow is detailed below.

For each test sample with a fixed SysML diagram category, the pipeline first constructs input prompts. The retrieval-augmented branch enriches the standard Type1_SimpleRule template with the most lexically similar same-category training sample as contextual reference, while the baseline only uses the bare standardized template without additional modeling examples.

Under unified inference hyperparameters, the model outputs raw PlantUML text. Code blocks wrapped by “@startuml” and “@enduml” are extracted as candidate model outputs. All syntax inspection is executed solely by the PlantUML parsing engine. Graphviz only renders graphical layouts for fully valid code and does not participate in syntax error detection or validity judgment.

If syntax defects or incomplete PlantUML delimiter tags are detected, detailed error logs are appended to the original prompt as corrective guidance for regeneration. Candidate code passing full grammatical inspection is directly retained as final output. If three correction attempts hit the preset upper limit, the latest generated code is reserved as the model output.

This error-driven closed-loop module effectively reduces structurally invalid SysML model outputs. The retrieval-augmented mode leverages verified same-category modeling examples to standardize SysML element structures and syntactic writing conventions, whereas the plain-prompt baseline reflects the native generation capacity of the QLoRA fine-tuned LLM. Separate statistics for one-pass native generation pass rate and post-correction final pass rate will be reported independently in the experimental chapter on the 131 held-out test set, which quantitatively disentangles performance improvements brought by model fine-tuning and context reference augmentation, supporting reliable ablation analysis.

## 3. Results

**Figure 10:**
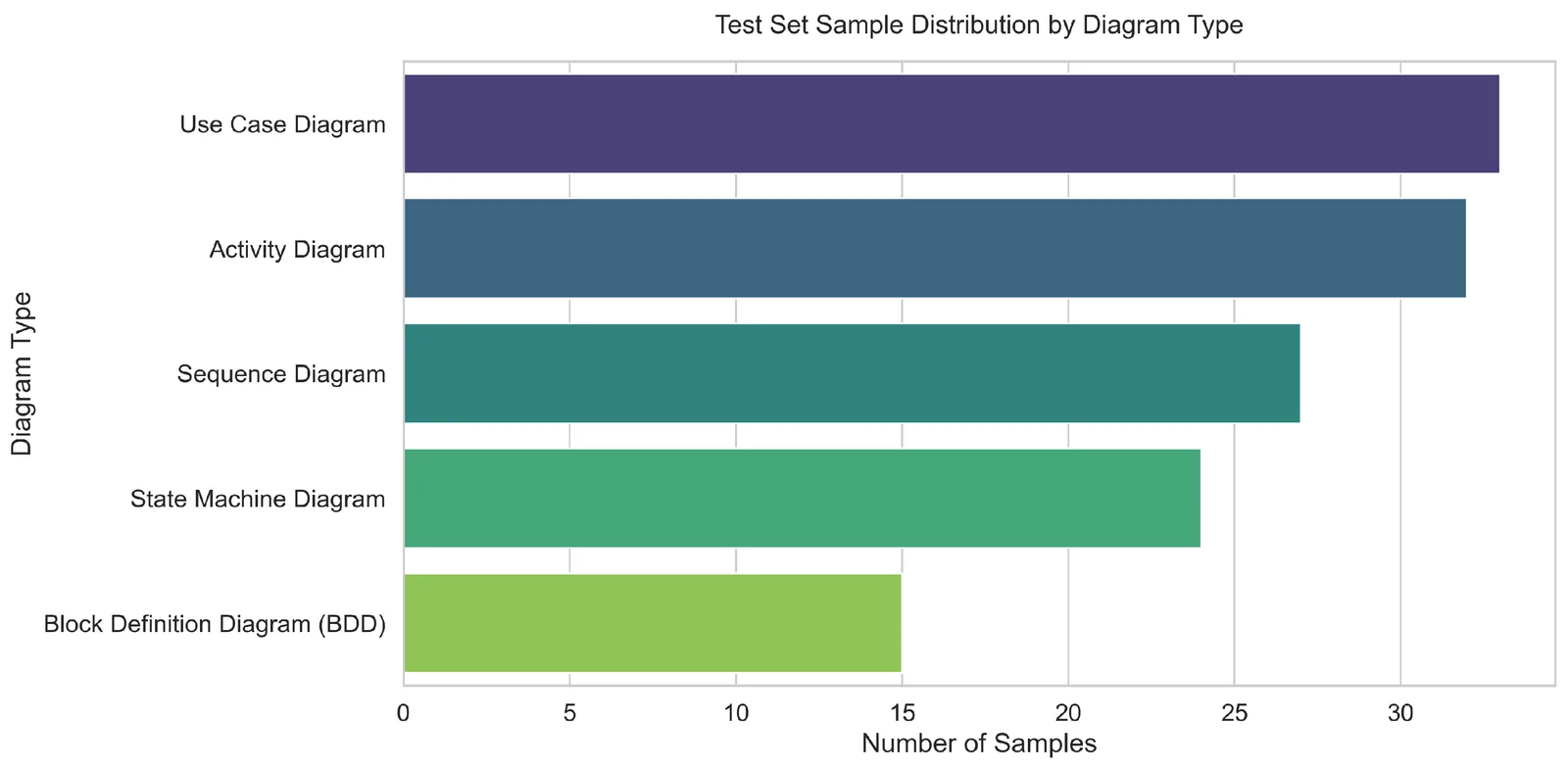
Test sample quantity distribution grouped by SysML diagram type.

All training and test samples used in this paper are pre-filtered via PlantUML syntax parsing to guarantee that all ground-truth SysML v1 PlantUML code is fully parseable. The held-out test set covers five mainstream SysML diagram categories: block definition diagrams (BDDs), use-case diagrams, activity diagrams, sequence diagrams, and state machine diagrams. Test samples are evenly distributed across five diagram types and three complexity tiers (simple, medium, complex), as visualized in Figure 10, supporting balanced, objective quantitative evaluation of generation performance. The test set contains 131 independent samples in total. SysML requirement diagrams and parametric diagrams are not included within the corpus scope.

Two inference pipelines derived from the fully offline proposed framework are compared via ablation analysis:

1. Baseline: Fixed Type1_SimpleRule prompt template without auxiliary reference PlantUML snippets.
2. Retrieval-Augmented: For each test input, offline Jaccard word-set similarity matching retrieves the most lexically similar training sample sharing an identical SysML diagram type as in-context reference.

Both pipelines integrate the identical closed-loop syntax correction module, with a hard upper limit of three regeneration retries per sample to avoid infinite iteration triggered by persistent syntax defects. The correction loop terminates early once generated code passes full PlantUML grammar inspection. If all three retry attempts are exhausted, the latest output is retained.

Two standardized quantitative metrics evaluate SysML generation quality, split into syntax-related and semantic matching indicators.

1. Final valid render rate: The share of samples whose output passes PlantUML parsing after iterative correction, reflecting overall format compliance of generated SysML models.
2. Word-set semantic F1: Core metric quantifying semantic alignment between generated PlantUML and ground-truth annotations. Calculation follows the intersection-union word-set rule defined in Section 2.2, with token order ignored during computation.

Two auxiliary derived indicators separate native model generation capacity and gains brought by post hoc correction. The zero-retry pass rate records the proportion of samples producing grammatically valid PlantUML in the first forward pass without error-guided regeneration. The final valid render rate measures overall format performance after multi-round repair. Since the closed-loop correction module artificially elevates syntax compliance, zero-retry pass rate and word-set semantic F1 are adopted as primary comparison metrics to fairly quantify performance gaps between baseline and retrieval-augmented schemes.

### 3.1. Overall Performance Comparison of Two Schemes

Table 5 aggregates global metrics for the baseline and Jaccard retrieval-augmented pipelines across the full 131-sample test set.

**Figure 11:**
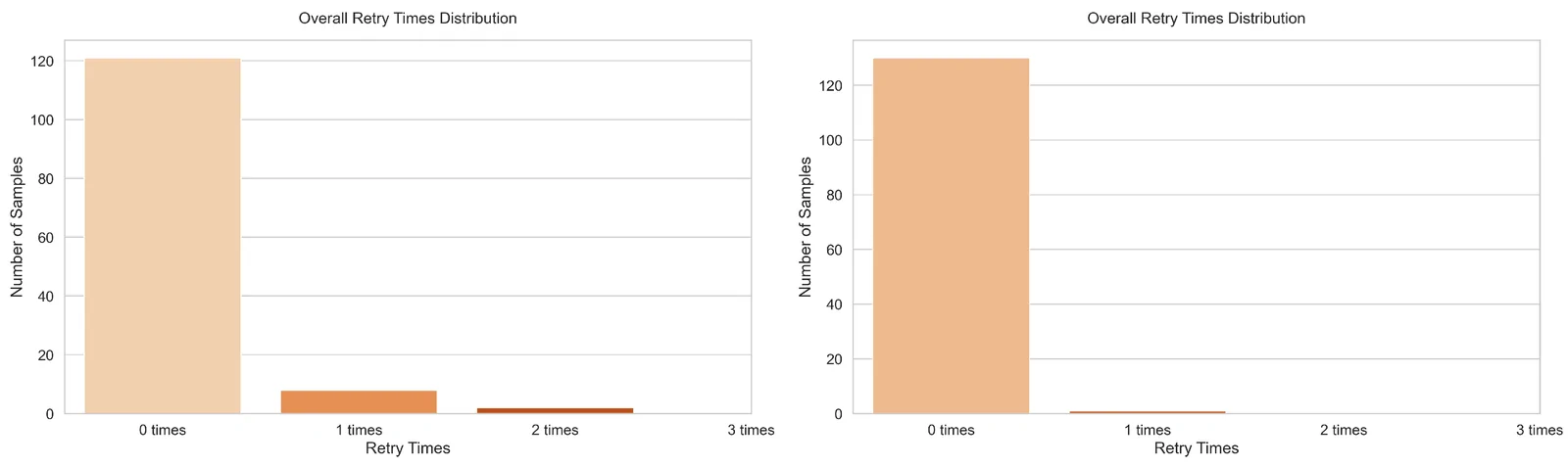
(**Left**): retry distribution of prompt-only baseline. (**Right**): retry distribution of retrieval-augmented dynamic example injection.

Global statistics show that the dynamic retrieval-augmented scheme lifts the zero-retry syntax pass rate substantially from 92.37% to 99.28% and cuts average correction retries to only 0.03. This demonstrates that category-matched reference snippets effectively guide the model to output syntax-compliant PlantUML in the initial generation and drastically reduce reliance on iterative error repair. The retry count distribution for both groups is illustrated in Figure 11.

In terms of semantic matching, the baseline achieves a marginally higher average word-set semantic F1. Although matched reference examples standardize overall SysML structural syntax, strict diagram-type constraints restrict the model’s flexible expression of diversified requirement logic, leading to a mild drop in overall semantic alignment.

Notably, the retrieval-augmented group fails to generate fully parseable code after three correction rounds for exactly one complex multi-branch sample, resulting in its 99.28% final valid render rate rather than 100%. Nested multi-way logic within this sample conflicts with fixed structural patterns imposed by reference examples, so repeated correction cannot produce valid PlantUML.

### 3.2. Per-Diagram-Type Performance Analysis

**Figure 12:**
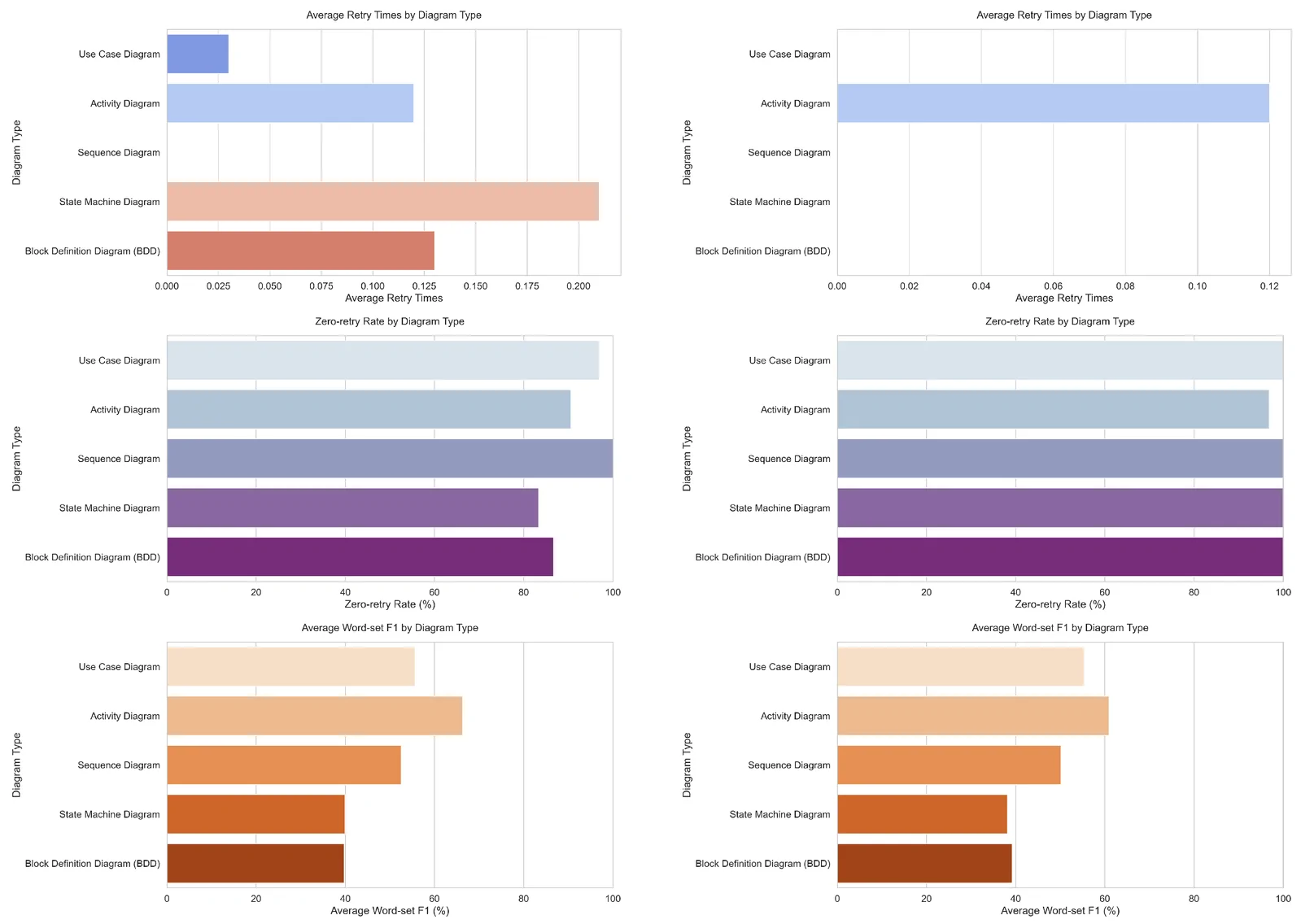
(**Top row**): average retries by diagram type (left: baseline, right: retrieval-augmented). (**Middle row**): zero-retry pass rate by diagram type (left: baseline, right: retrieval-augmented). (**Bottom row**): word-set semantic F1 by diagram type (left: baseline, right: retrieval-augmented).

Performance gaps between the two pipelines differ visibly across the five SysML diagram categories. Test sample counts grouped by diagram type are shown in Figure 10. Four core indicators are recorded per category: final valid render rate, average retry times, zero-retry pass rate, and word-set semantic F1. Comparative visualizations are presented in Figure 12.

Across all five SysML diagram types, the retrieval-augmented pipeline consistently reduces average correction retries and raises the zero-retry syntax pass rate. The performance gain is most prominent for activity and sequence diagrams, which contain intricate entity associations and interactive logic. In contrast, the baseline maintains higher semantic F1 across all categories, as it is not bounded by rigid structural patterns derived from reference examples.

### 3.3. Performance Under Different Complexity Levels

**Figure 13:**
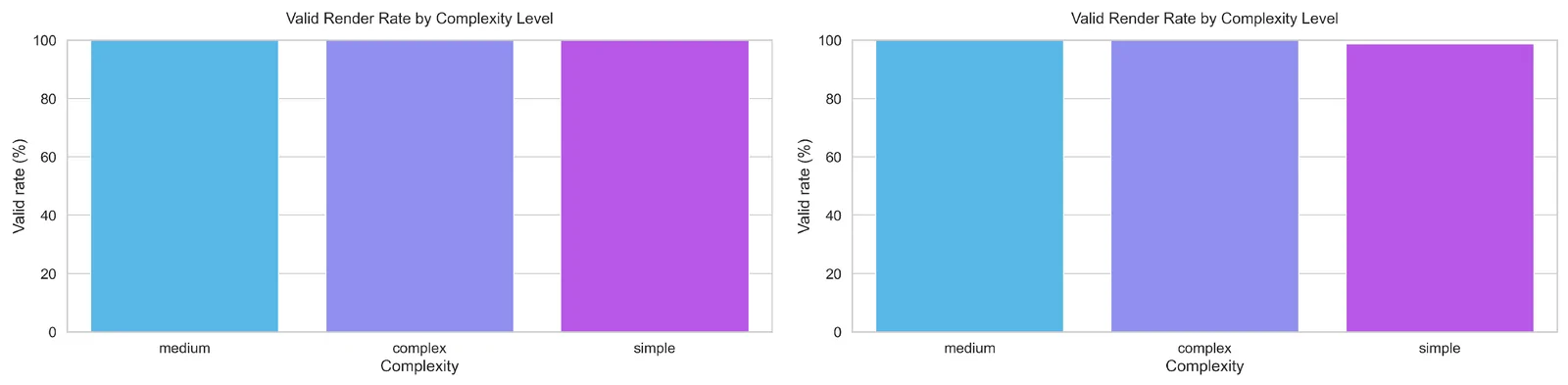
(**Left**): valid render rate grouped by complexity tier (baseline). (**Right**): valid render rate grouped by complexity tier (retrieval-augmented).

Test samples are split into simple, medium, and complex subgroups by modeling complexity. Both pipelines achieve nearly full valid render rates across all complexity tiers, as shown in Figure 13. The retrieval-augmented scheme retains lower retry overhead at every complexity grade, proving that dynamically matched SysML reference examples stabilize generation quality regardless of system logic scale.

### 3.4. Word-Set Semantic F1 Distribution of All Test Samples

**Figure 14:**
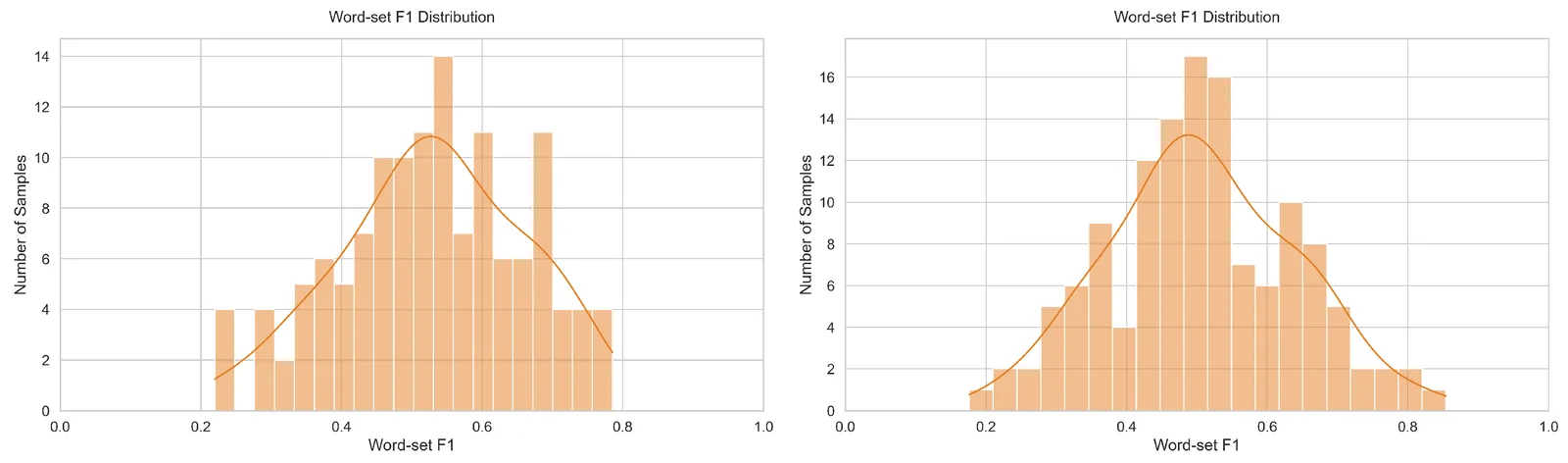
(**Left**): word-set semantic F1 distribution, baseline. (**Right**): word-set semantic F1 distribution, retrieval-augmented scheme.

Per-sample F1 distributions further reflect semantic matching stability for the two pipelines. Comparative statistical curves are provided in Figure 14. The baseline’s overall distribution shifts slightly toward higher F1 values, consistent with the global average semantic metric reported in Table 5.

### 3.5. Summary of Experimental Findings

Multi-dimensional comprehensive evaluation reveals clear performance trade-offs introduced by the offline Jaccard dynamic retrieval module for SysML v1 PlantUML generation:

1. The retrieval module substantially improves native single-pass syntax correctness and reduces reliance on iterative grammar correction.
2. Fixed-category reference examples impose mild structural constraints, leading to a slight decline in word-set semantic F1.
3. The embedded closed-loop correction mechanism delivers nearly fully parseable outputs for both pipelines on most samples, including complex multi-branch SysML models.
4. The performance advantage of dynamic reference retrieval holds steadily across all five SysML diagram types and all three complexity grades.

## 4. Discussion

Systematic reviews targeting automated SysML PlantUML generation point out two core bottlenecks restricting the development of this field: the lack of high-quality fully parseable domain datasets and the inherent trade-off among semantic generation capability, industrial data security constraints and hardware overhead for offline deployment. To address the above challenges, this paper constructs a grammar-screened paired corpus consisting of natural language requirements and standard SysML PlantUML code, and proposes a fully offline lightweight large language model framework with three core functional modules: 4-bit NF4 QLoRA domain fine-tuning, dynamic same-diagram reference retrieval based on Jaccard word-set similarity, and multi-round iterative PlantUML syntax correction.

It is necessary to clarify the modeling scope in advance. The generated PlantUML code complies with SysML v1 specifications and covers five mainstream structural and behavioral diagram types. SysML-exclusive requirement diagrams and parametric diagrams are not included in the corpus and the support range of the proposed model. Although the word-set semantic F1 of the lightweight 7B local model cannot reach the performance of trillion-parameter cloud commercial large language models, the complete offline execution mode avoids uploading confidential design data to third-party platforms. Meanwhile, this work fills the long-term vacancy of standardized, render-verified SysML training data for academic research and industrial practice.

### 4.1. Trade-Off Analysis of Dynamic Retrieval-Augmented Prompt Enhancement

Two inference pipelines are designed for ablation comparison to quantify the performance influence brought by the offline Jaccard retrieval module:

1. Baseline Prompt Only: Fixed Type1_SimpleRule template without supplementary reference PlantUML snippets.
2. Jaccard Dynamic Retrieval-Augmented In-Context Reference: For each test sample, the most lexically similar training sample with the identical SysML diagram category is dynamically retrieved, and its valid PlantUML code is appended to the baseline prompt as context reference.

All aggregated ablation indicators are summarized in Table 5. Dynamically injected reference examples matched by diagram type significantly stabilize native one-pass generation performance. The zero-retry syntax pass rate rises from 92.37% to 99.28%, and the average number of correction retries per sample drops from 0.09 to merely 0.03. Reference samples with consistent diagram types provide definite structural and syntactic constraints for SysML modeling, which effectively suppress random grammatical errors in raw model output.

Nevertheless, category constraints introduced by retrieved reference snippets limit the model’s flexible expression of diversified requirement logic, resulting in a slight decrease in the average word-set semantic F1 from 52.94% to 50.64%. The per-sample F1 distribution shown in Figure 14 further visualizes this trade-off relationship between syntax compliance and semantic flexibility. It is worth noting that both pipelines achieve a nearly 100 percent valid render rate after iterative error repair, which proves that the closed-loop correction module rather than prompt optimization dominates the final format compliance of generated code.

### 4.2. Comparison Between LLM Offline Framework and Traditional Rule-Based SysML Modeling

This paper systematically compares the end-to-end LLM-based scheme proposed in this work with classic rule-driven SysML generation methods, a mainstream two-category classification adopted by existing domain surveys. Rule-based modeling frameworks depend on manually compiled expert rules and require engineers to convert free-text natural language requirements into rigid structured descriptions before transformation execution [7,9,37]. In contrast, transformer-based large language models pre-trained on massive general text corpora directly accept unstructured natural language input, eliminating manual rule compilation and requirement normalization work [38,39]. Multi-dimensional comparative results of the two technical routes are listed in Table 6.

The nearly saturated renderable valid rate obtained in this paper is derived from the post-processing grammar verification loop instead of the native generation capability of the large language model. Syntax inspection is fully completed by the native PlantUML parser, and Graphviz only performs graphical layout rendering for fully valid code, which is consistent with the standard SysML v1 PlantUML workflow described in the existing literature. Different from multiple comparative studies that calculate F1 based on corrected valid output, this paper adopts token-level raw word-set overlap statistics to compute semantic F1, avoiding artificially inflated evaluation scores caused by multi-round error repair. Inconsistent statistical standards of semantic indicators across published papers hinder fair horizontal performance comparison between different algorithms, which reveals the necessity of unified standardized evaluation criteria for this research field.

### 4.3. Offline Lightweight LLM Versus Cloud Commercial Large Models

Most existing empirical evaluations of cloud commercial large language models adopt the small-scale ACM107 corpus, which only covers three behavioral SysML diagram types [17]. The test category distribution, prompt design and complete evaluation pipeline differ greatly from the setup of this paper, which supports five types of structural and behavioral SysML diagrams. Therefore, the reported performance of cloud models cannot be directly used for fair horizontal comparison with the offline framework proposed in this work.

All mainstream ultra-large foundation models are only available through cloud API services. Enterprises have to upload confidential internal system design requirements to third-party servers, which brings irreversible risks of proprietary design information leakage [12]. In addition, trillion-parameter foundation models require huge high-end GPU memory resources, making local domain fine-tuning and offline customized deployment infeasible for most small and medium-sized engineering teams [40,41].

According to statistics summarized in the relevant literature, the overall SysML generation F1 of existing hybrid rule–Natural Language Processing (NLP) pipelines ranges only from 70.2% to 74.4%, while term-extraction-oriented approaches reach as low as 33.3% in semantic matching metrics. It should be noted that the calculation rules of these indicators do not uniformly adopt the word-set intersection-union standard defined in this paper, so the comparison can only reflect approximate performance gaps. Although cloud large language models possess stronger general semantic comprehension capability trained on massive universal text corpora, they cannot satisfy the offline secure modeling requirements of confidential industrial system design scenarios.

This paper applies 4-bit NF4 quantization to Qwen2.5-Coder-7B to realize full local fine-tuning and inference on a single A10 GPU without Internet access. The low hardware threshold supports low-cost offline deployment for small engineering teams, which is a core practical advantage not possessed by cloud-exclusive proprietary model solutions. Balancing deployment cost, data privacy and modeling quality, this offline framework provides a feasible alternative for confidential SysML modeling tasks.

### 4.4. Core Contribution: Construction of High-Quality SysML Domain Corpus

The most prominent independent contribution of this work lies in constructing a large-scale corpus dedicated to SysML v1 PlantUML generation with full grammar verification, which fills the standardized dataset gap repeatedly pointed out in recent domain review papers.

**Figure 15:**
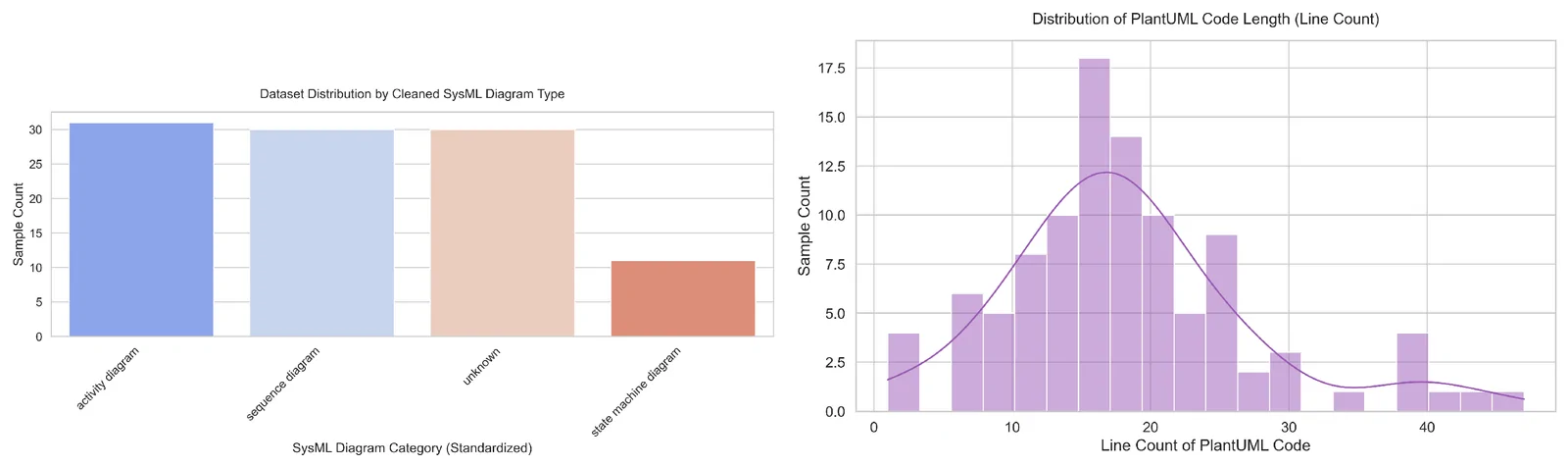
(**Left**): diagram type distribution of original ACM107 dataset. (**Right**): ground-truth PlantUML code length distribution of ACM107 dataset.

At present, the ACM107 dataset proposed by Wang et al. [17] is the publicly available corpus for this research field, yet it has obvious defects in sample quantity, category coverage and data quality. Its original data pool only contains 102 raw entries, and merely 70 parseable samples are retained after filtering, covering three behavioral SysML diagram types only. Figure 15 reflects its uneven category distribution and narrow coverage of code complexity. Most other published research does not open training data to the public, while the public PlantUML corpus on HuggingFace contains a large number of unfiltered invalid code that cannot be used for formal model training.

By contrast, all samples in the corpus constructed in this paper pass dual inspection of PlantUML syntax and rendering to eliminate unparseable invalid modeling fragments. The corpus covers five standard SysML diagram types, with evenly distributed samples across three complexity grades of simple, medium and complex, forming reliable mapping pairs between free-text industrial requirements and standard renderable PlantUML. Corresponding overall statistical indicators can be found in Table 2. Compared with the incomplete small-scale ACM107 dataset, this corpus contains richer industrial modeling scenarios, complete coverage of structural and behavioral diagrams, and stricter data quality control standards. Even though the lightweight offline model cannot reach the semantic F1 level of trillion-parameter cloud large language models, the high-quality annotated corpus itself can provide reusable standardized research resources for follow-up automated SysML modeling studies.

### 4.5. Comparison with Existing LLM Hallucination Suppression Routes

Existing research has proposed hybrid rule–NLP pipelines, multi-agent collaborative modeling and multi-round feedback loops to mitigate semantic hallucination of large language models [15,42]. However, most multi-agent architectures are customized for SysML v2 specifications and cannot be directly migrated to the SysML v1 workflow compatible with PlantUML adopted by mainstream lightweight industrial drawing tools. A variety of general hallucination suppression strategies have not been fully explored and adapted to SysML modeling scenarios [23,24].

Different from computationally intensive multi-agent frameworks, this paper adopts a lightweight pipeline combining dynamic retrieval prompt enhancement and iterative syntax correction. It achieves lower inference delay and better deployability for offline industrial SysML modeling tasks.

### 4.6. Intrinsic Limitations of the Proposed Framework

The generation performance of domain large language models is fundamentally restricted by the lack of standardized render-verified domain datasets [21,22]. Although this work constructs a fully filtered dedicated corpus, several inherent limitations consistent with common challenges of this field remain to be further optimized:

1. Limited sample scale and unbalanced logical distribution. Despite having a larger volume than the public ACM107 corpus, the overall corpus size is still constrained. Samples containing multi-branch complex business logic account for less than seven percent of valid data, which reduces the semantic matching F1 on state machine diagrams and block definition diagrams with intricate logical relations.
2. Generalization risk caused by homologous data source. Training and test samples are collected from homologous industrial requirement scenarios, without independent external cross-domain validation sets. No quantitative measurement of inter-set similarity is implemented, which weakens the reliability of generalization performance on unseen heterogeneous system design demands.
3. Single-dimensional evaluation system. Current evaluation only relies on syntax parse validity and token-level word-set semantic F1, lacking quantitative indicators to measure the logical rationality, structural integrity and practical engineering value of generated SysML diagrams.

### 4.7. Future Research Directions

Three targeted improvement paths are proposed to solve the above three limitations, with clear executable technical routes corresponding to each defect:

1. Expand the scale of high-quality domain corpus. Enrich the total data volume and supplement sufficient multi-branch complex modeling samples through industrial requirement collection and LLM-based data augmentation. This measure can narrow the semantic performance gap with ultra-large cloud foundation models and balance the uneven distribution of complex logic samples.
2. Introduce lightweight SysML-oriented hallucination suppression strategies. Adapt low-overhead semantic error mitigation mechanisms to the offline pipeline of SysML v1 combined with PlantUML to reduce logical contradictions in generated diagram structures.
3. Establish multi-dimensional standardized domain evaluation criteria. Construct a comprehensive quantitative assessment system covering syntax standardization, structural rationality and engineering practicability, matched with standardized expert scoring rules, to realize rigorous multi-angle measurement of automated SysML generation quality.

To summarize, this work constructs a dedicated high-quality SysML PlantUML corpus and a fully offline lightweight 7B large language model generation framework based on QLoRA fine-tuning, dynamic Jaccard retrieval-augmented in-context learning and closed-loop syntax correction. The proposed scheme balances offline data security, low deployment cost and basic SysML modeling capability and reveals the trade-off relationship between native syntax correctness and semantic flexibility brought by category-matched reference retrieval. The summarized dataset defects and single-dimension evaluation limitations point out clear improvement directions for follow-up research on automated SysML diagram generation.

## 5. Conclusions

Traditional rule-based SysML modeling heavily relies on manually engineered expert transformation rules and mandatory structured rewriting of raw requirements, suffering from weak cross-domain generalization and substantial manual preprocessing overhead. Although pre-trained large language models mitigate these drawbacks, direct LLM-driven SysML PlantUML generation still faces three critical industrial bottlenecks. First, the absence of syntax-validated domain-specific datasets caps upper generation performance. Second, inherent semantic and syntactic hallucinations cannot be fully eliminated, while SysML-customized suppression strategies remain underdeveloped. Third, commercial cloud LLM services impose irreversible leakage risks on confidential industrial design specifications.

To address the above academic and practical challenges, this paper delivers two major contributions. From the data perspective, we construct a large-scale paired corpus linking natural language requirements and standard SysML v1 PlantUML code, with all samples pre-screened via PlantUML syntax inspection to guarantee fully parseable ground-truth artifacts. From the algorithm perspective, we propose a fully offline lightweight LLM generation framework built upon three core modules: 4-bit NF4 QLoRA domain fine-tuning, dynamic same-category reference retrieval via Jaccard word-set similarity, and multi-round iterative closed-loop syntax correction.

The complete pipeline runs locally on a single consumer-grade A10 GPU without transmitting confidential enterprise design data to third-party cloud platforms, fundamentally eliminating data security hazards. During inference, the offline retrieval module dynamically injects validated category-matched SysML reference snippets to standardize output syntax and suppress native grammatical hallucinations. Combined with a post-inference error-feedback correction loop, the framework achieves nearly fully renderable outputs after iterative repair.

Quantitative ablation experiments on a 131-sample held-out test set covering five mainstream SysML v1 diagram types reveal a clear performance trade-off introduced by retrieval-augmented context injection. Matching reference examples sharing identical diagram categories substantially boost the one-pass zero-retry syntax pass rate and cut average correction retries, yet rigid structural constraints slightly reduce word-set semantic F1 by limiting flexible expression of diverse requirement logic. The optimization gain brought by retrieval enhancement remains consistent across simple, medium, and complex modeling samples.

Compared with the publicly available ACM107 dataset, which features a small sample volume and incomplete diagram coverage, our self-built corpus contains richer industrial modeling scenarios, balanced multi-type distribution, and stricter syntax filtering criteria, serving as standardized reusable training resources for follow-up research. The proposed offline lightweight scheme balances deployment cost, data privacy, and generation quality, delivering a viable technical route for confidential industrial requirement engineering and automated SysML modeling.

It should be noted that the framework still carries inherent limitations, including insufficient multi-branch complex samples and a single-dimensional evaluation system, which are elaborated thoroughly in the Discussion section.

Three targeted improvement directions are proposed to resolve the identified limitations: first, expand the scale of the high-quality domain corpus. Additional multi-branch complex modeling samples will be supplemented through industrial requirement collection and LLM-based data augmentation to balance sample distribution and narrow the semantic performance gap with ultra-large cloud foundation models. Second, embed lightweight SysML v1/PlantUML-tailored semantic hallucination suppression mechanisms into the offline inference pipeline to reduce logical contradictions within generated diagram structures. Third, construct a multi-dimensional comprehensive evaluation system covering syntax standardization, structural rationality, and engineering practicability, enabling full quantitative measurement of automated SysML generation quality.

In summary, this work constructs a dedicated high-quality SysML PlantUML corpus and a fully offline lightweight 7B LLM generation framework integrating QLoRA fine-tuning, Jaccard retrieval-augmented in-context learning, and closed-loop syntax correction. The scheme strikes a practical balance between offline data security, low hardware overhead, and baseline SysML modeling capacity, and the revealed trade-off between native syntax compliance and semantic flexibility provides clear guidance for future research on secure automated system modeling.

## Figures and Tables

**Table 1:** Comparison of representative rule-based SysML generation approaches.

Approach	Supported SysML Diagrams	Dataset Scale	Core Evaluation Means
ROM-based transformation [7]	BDD, use case, activity	Small self-manually built corpus	Manual semantic comparison
RNL2SysML [8]	Activity, control-oriented BDD	Aircraft industrial case data	Domain expert qualitative scoring
Structured template rule pipeline [9]	BDD, modular subsystem diagrams	Small custom domain corpus	Qualitative case verification
Rule–ML hybrid refinement pipeline [10]	General structural diagrams	Unpublished industrial requirement texts	Iterative expert feedback adjustment

**Table 2:** Overall basic statistics of the constructed SysML modeling dataset.

Statistical Metric	Specific Value
Total valid SysML PlantUML paired samples	914
Covered independent industrial business domains	16
Included standard SysML v1 diagram categories	5
Simple-complexity samples	497
Medium-complexity samples	234
Complex-complexity samples	183
Full sample syntax and rendering pass rate	100.00%
Training set size	783
Test set size	131

**Table 3:** Performance of seven baseline prompt variants (Stage 1 ablation, 20 test subset samples).

Prompt Type	Precision	Recall	Word-Set Semantic F1	Parse Pass Rate
Type1_NoSysUser	0.5594	0.3513	0.4142	55.00%
Type2_TrainFormat	0.5582	0.3665	0.4303	65.00%
Type3_Simple	0.5860	0.3562	0.4309	65.00%
Type4_FormatRule	0.6502	0.3336	0.4265	75.00%
Type5_CoT	0.5134	0.3504	0.3838	50.00%
Type6_ExpertRole	0.6077	0.3606	0.4348	65.00%
Type7_FullConstraint	0.6029	0.3324	0.4125	65.00%

**Table 4:** Performance of two refined prompt templates (Stage 2 ablation, 20 test subset samples).

Prompt Type	Precision	Recall	Word-Set Semantic F1	Parse Pass Rate
Type1_SimpleRule	0.5762	0.3702	0.4378	75.00%
Type2_ExpertDiagramRule	0.6058	0.2975	0.3881	60.00%

**Table 5:** Global aggregated evaluation metrics of two inference strategies.

Metric	Baseline Prompt Only	Retrieval-Augmented
Total test samples	131	131
Final valid render rate	100.00%	99.28%
Average retry times per sample	0.09	0.03
Zero-retry pass rate	92.37%	99.28%
Average word-set semantic F1	52.94%	50.64%

**Table 6:** Comprehensive comparison between rule-based and LLM-driven SysML generation approaches.

Evaluation Dimension	Rule-Based Methods	Offline LLM Framework
Input format	Structured constrained natural language	Unrestricted free natural language
Knowledge source	Manual expert rules	Annotated requirement-PlantUML dataset
Syntax determinacy	Fully fixed under standard input	Uncertain, prone to modeling hallucination
Cross-domain scalability	Low, tightly bound to specific industrial domains	High, learns universal linguistic patterns
Manual preprocessing burden	Heavy mandatory requirement rewriting	Low, end-to-end automatic mapping
Hardware deployment cost	Minimal computing resource demand	Medium, operable on single consumer-grade GPU
Data leakage risk	None, fully local execution	None, complete offline inference without cloud access

## Data Availability

The public raw PlantUML corpus used as the basic data source of this study can be accessed at https://www.kaggle.com/datasets/therayto/sysml-text-plantuml-914 (accessed on 10 June 2026). The small-scale ACM107 comparative dataset proposed by Zhao et al. can be acquired through the Google Drive at https://drive.google.com/drive/folders/10eo8KDqlBlkQZxPpPCB7R3-aBQZ7Rsm6?usp=drive_link (accessed on 10 June 2026). The high-quality grammar-filtered SysML-PlantUML paired dataset constructed in this paper, which contains 914 valid samples covering five categories of SysML v1 diagrams, will be made publicly available together with the fixed train/test split scheme, random seed and full data cleaning filtering rules after the manuscript is accepted for publication.

## Abbreviations

The following abbreviations are used in this manuscript:

SysML	Systems Modeling Language
MBSE	Model-Based Systems Engineering
BDD	Block Definition Diagram
LLM	Large Language Model
NLP	Natural Language Processing
LoRA	Low-Rank Adaptation
QLoRA	Quantized Low-Rank Adaptation
PEFT	Parameter-Efficient Fine-Tuning
SFT	Supervised Fine-Tuning
NF4	4-bit normalized floating-point quantization
GPU	Graphics Processing Unit
VRAM	Video Random Access Memory
API	Application Programming Interface
